# Experimental menopause in 3xTg-AD mice exacerbates metabolic, inflammatory, and osteologic phenotypes aligned with Alzheimer’s disease pathology

**DOI:** 10.21203/rs.3.rs-7769003/v1

**Published:** 2025-10-06

**Authors:** Jessica L. Dennison, Maggie A. Miller, Aikta Sharma, Ava M. Cherry, Irina Djuraskovic, J. Paul Chapple, James A. Timmons, Andrew A. Pitsillides, Claes Wahlestedt, Claude-Henry Volmar

**Affiliations:** University of Miami Miller School of Medicine; University of Miami Miller School of Medicine; University College London; University of Miami Miller School of Medicine; University of Miami Miller School of Medicine; Queen Mary University of London; Queen Mary University of London; Royal Veterinary College; University of Miami Miller School of Medicine; University of Miami Miller School of Medicine

**Keywords:** Menopause, Alzheimer’s Disease, Periphery, Inflammation, Osteoporosis, Insulin resistance

## Abstract

**Background:**

Alzheimer’s disease (AD) is neurodegenerative disease characterized by the accumulation of amyloid-beta plaques and phosphorylated tau. An estimated 7.2 million Americans are currently living with AD, nearly two-thirds of which are women. Sex differences in AD prevalence and pathology are well established, however the mechanisms underlying these differences are understudied. There are compelling links between menopause and AD, but few established common molecular mechanisms partly due to the lack of representative experimental models.

**Methods and results:**

Here, we induce an accelerated ovarian failure (OF) model of menopause in the triple-transgenic AD (3xTg-AD) mouse, using ovotoxin 4-vinylcyclohexene diepoxide (VCD) mediated follicular depletion, leading to a loss of circulating progesterone and an increase in plasma follicle-stimulating hormone (FSH) levels—hormonal changes that closely mirror those observed in human menopause. OF exacerbated peripheral phenotypes associated with AD, namely insulin resistance, inflammation, and bone mass and architecture modifications resembling osteoporosis. OF aggravated age-related impaired glucose tolerance and caused insulin resistance. Additionally, plasma levels of four proinflammatory cytokines- IL-5, IL-6, TNF-α, and CXCL- were all increased in OF mice compared to non-menopausal AD mice. Meanwhile, OF mice display heightened bone loss phenotype, a condition with known links to AD risk and pathology.

**Conclusion:**

In summary, accelerated ovarian failure presents key metabolic, inflammatory, and skeletal phenotypes associated with AD, indicating that it can be useful for the identification of novel therapeutic targets.

## Introduction

Alzheimer’s disease (AD) is a neurodegenerative disease characterized by extracellular amyloid-beta (Aβ) and intracellular neurofibrillary tau tangles, accompanied by brain inflammation and atrophy (1). It is a multifaceted disease, with high levels of inter-patient variability in pathology making it challenging to develop successful treatments (2). Women have a disproportional risk of AD, nearly twice that of men, with prevalence doubling over the age of 70 (1, 3, 4). The importance of sexual dimorphism in AD pathophysiology is gaining attention, not least because women also appear to demonstrate more severe pathology (5), including greater Aβ (6, 7) and tau (8, 9) burden (10). Development of new models which capture the influence of sexual dimorphism, coupled with molecular profiling may help identify druggable pathways which drive this accelerated risk, as well as help with the evaluation of suitable biomarkers or prognostics which identify women most at risk.

One hypothesis for the greater prevalence of AD in women is that it is caused by the extreme *fluctuations* in sex hormones experienced as they traverse menopause and undergo the *depletion* of estrogen and progesterone (11). Although the end result of menopause is reproductive senescence, the consequential changes in sex hormones impair many physiological systems including sleep disturbances, depression, and cognitive impairment (12). Indeed, menopause has been reported to affect both learning and memory in humans, as well as in rodent models (13–16). Notably, menopause is accompanied by the loss of neuromuscular metabolic gene expression—a hallmark of aging (17)—and impaired glucose metabolism, both of which are potential contributors to AD pathology and worsened clinical outcomes (18). The menopausal transition is also linked to heightened central and peripheral inflammation and increased risk of osteoporosis, all of which are associated with greater AD risk and progression(17–20).

Menopause is challenging to study in humans as large inter-subject variation requires large cohorts to adequately model the disease processes. Establishing a preclinical model that features pathway level interactions between AD, age, and menopause would be of substantial utility. While rodents do not go through menopause *per se*, surgical removal of the ovaries (ovariectomy) represents one, albeit abrupt, model of estrogen loss. While studies show that the abrupt loss of gonadal hormones in rodents results in behavioral abnormalities, bone loss and an exacerbation of AD pathology(21), the ovariectomy model comes with significant limitations (22–24). Notably, the ovariectomized model cannot simulate the menopausal transition through perimenopause, a state of extreme hormonal variation, during which women often first complain of cognitive deficits. This potentially critical phase has therefore not been studied (12), and it may well be the most important timeframe to intervene with novel drug treatments aimed at reducing risk of AD.

Inducing progressive ovarian failure via injection of the ovotoxin 4-vinylcyclohexene diepoxide (VCD) represents a recent and novel strategy to mimic the human menopause (25). Accelerated ovarian failure (OF) using chronic, low-doses of VCD better reproduces human menopause for a number of reasons (25). First, follicles are lost gradually through apoptosis while maintaining ovarian tissue, consistent with the human condition (26). Second, levels of estrogen, progesterone, follicle stimulating hormone (FSH), and luteinizing hormone (LH) fluctuate, ending at levels similar to that observed in human menopause (27). Importantly, this model allows for representation of pre-menopause, early and late perimenopause, and post-menopause. In the present study we explore the links between menopause and AD by evaluating the impact of VCD induced ovarian failure on several peripheral systems associated with AD risk and pathology in the 3xTg-AD mouse. We established that ovarian failure in the 3xTg-AD mouse negatively impacts peripheral metabolic health while simultaneously increasing peripheral inflammation and accelerating osteoporosis-like bone loss phenotypes. This first report on a VCD menopause model in the 3xTg-AD mouse adds to the growing resources that enable the study of the links between menopause and AD.

## Methods

### Study Approval and Sex as a biological variable

All experiments were approved by the University of Miami Miller School of Medicine Institutional Animal Care and Use Committee (IACUC). Our study exclusively examined female mice because menopause is considered only relevant in females.

### Animals and Chemical Treatment

Female triple-transgenic AD (3xTg-AD)(28) mice were purchased through The Jackson Laboratory and the NIH supported Mutant Mouse Resource and Research Center (MMRRC). Mice were housed four animals per cage in an AAALAC-accredited animal facility at the University of Miami Miller School of Medicine. Ovotoxin vinylcyclohexene dioxide (VCD; Sigma, cat. #94956) was diluted in sesame oil (Fisher Scientific, cat #18–606-186). Animals were administered 160 mg/kg VCD (*n* = 19 for cohort 1, *n =* 30 for cohort 2) to induce OF or sesame oil vehicle (*n* = 20 for cohort 1, *n =* 30 for cohort 2) at a final volume of 100μL intraperitoneal (i.p.) starting at 4 months of age. Mice were injected 5 days a week for 3 weeks.

### Timeline

**Figure 1:**
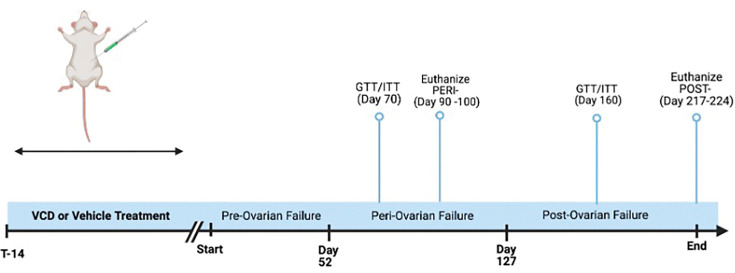
Timeline of treatment and menopause stages.

Based on prior studies using VCD to induce OF, early peri-OF begins at 52 days post first injection (d.p.i.), late peri-OF occurs around 73 d.p.i., and post-OF begins around 127 d.p.i. (29, 30). Behavioral testing was performed on peri-OF mice 53–68 d.p.i. (*n* = 39), when mice were 25–26 weeks old (Fig. 1). Glucose tolerance testing (GTT) for peri-OF was performed at 70–71 d.p.i., (*n* = 39) when mice were 27 weeks old. Mice representing peri-OF (Ctrl *n* = 9; VCD *n* = 8) were euthanized 90–100 d.p.i., when mice were 30–32 weeks old. GTT for post-OF mice (*n* = 20) was performed at 154 d.p.i., when mice were 39 weeks old. Insulin tolerance testing (ITT) was performed at 160 d.p.i., or 40 weeks of age. Mice representing post-OF (Ctrl *n* = 10; VCD *n* = 10) were euthanized 217–224 d.p.i., approximately 48 weeks old. A second cohort of mice was used following the same timeline as the first cohort but utilizing a larger sample size (peri-vehicle *n* = 15, peri-VCD *n* = 15, post-vehicle *n* = 15, post-VCD *n* = 15).

### Glucose and Insulin Tolerance

Mice underwent intraperitoneal (i.p.) GTT at 6 and 9 months of age, and i.p. ITT at 9.5 months of age. For both GTT and ITT, mice were food fasted 6 hours prior to testing, with access only to water. For GTT, mice were injected i.p. with 1.5 g/kg body weight glucose diluted in saline and blood glucose was measured by a glucometer (Bayer) at 0, 15, 30, 60, 90, and 120 min. For ITT, insulin was given at 0.5 U/kg body weight i.p. and blood glucose was measured at 0, 15, 30, 45, 60, and 90 min.

### Tissue and Bone Collection

Mice from cohort 1 were anaesthetized with isoflurane and blood was collected by transcardial puncture at 7 months age (for PERI-OF) and 12 months age (for POST-OF). Mice from cohort 2 were anaesthetized with isoflurane and euthanized via perfusion with cold 1X PBS at 7 months of age (for PERI-OF) and 12 months of age (for POST-OF). Blood was collected with EDTA and stored on ice and then centrifuged at 2,000 × *g* at 4°C for 10 min for plasma and stored at −80°C until use. Left hindlimbs were removed from each mouse and fixed in 10% neutral buffered formalin overnight then washed in 1X PBS before transfer to 70% EtOH and stored at 4°C until micro-computed tomography. The brain was extracted and quickly dissected on ice to collect prefrontal cortex (PFC), hippocampus, entorhinal cortex (ERC), thalamus region, and cortex, which were immediately frozen on dry ice. A piece of liver was extracted and immediately frozen. Frozen samples were stored at −80°C until processed for RNA or protein extraction.

### Ovary histology and follicle counting

At the time of tissue collection, the left ovary was placed in Bouin’s fixative for 2 hours before being transferred to 70% ethanol and subsequently paraffin-embedded. Embedded ovaries were sectioned at 5 μm thickness and mounted every 20 sections to avoid double counting follicles. Ovary slices were stained with hematoxylin and eosin (H&E; H&E Staining Kit, Abcam, ab245880) and primordial (oocyte surrounded by single layer of flattened pre-granulosa cells), primary (oocyte surrounded by a complete layer of cuboidal granulosa cells), secondary (oocyte surrounded by multiple layers of granulosa cells), and antral (follicles containing a fluid-filled antrum) follicles were counted based on prior studies (31).

### ELISA

Ovarian hormones, estradiol and progesterone, and pituitary hormones, LH and FSH, were measured in mouse plasma using ELISA. Estradiol was measured using 17β-Estradiol high sensitivity ELISA kit (Enzo Life Sciences, cat. # ADI-900–074) at a 1:2 dilution. Progesterone was measured using Mouse/Rat Progesterone ELISA kit (MyBioSource, MBS495057). LH was measured using Mouse LH ELISA kit (MyBioSource, MBS2514287) at a 1:4 dilution. FSH was measured using Mouse follicle stimulating hormone (FSH) ELISA kit (ABclonal Technology, RK04237) at a 1:100 dilution. Each ELISA was performed per manufacturer instructions and measured using the EnVision^®^ multimode plate reader (Perkin Elmer).

### MSD Cytokine Multiplex Assay

Ten proinflammatory cytokines—IFN-γ, IL-1β, IL-2, IL-4, IL-5, IL-6, IL-10, IL-12p70, KC/GRO, and TNF-α—were quantified using a chemiluminescence-based assay from Meso Scale Discovery (MSD, Gaithersburg, MD, USA). Undiluted plasma samples were analyzed in duplicate using the V-PLEX Proinflammatory Panel 1 Mouse Kit (MSD, catalog # K15048D) following the manufacturer’s protocol. Plates were read on the MESO QuickPlex SQ 120 instrument, and cytokine concentrations were calculated based on standard curves generated with MSD DISCOVERY WORKBENCH^®^ 4.0 software. The assay detection limits for all ten analytes were in the picogram per milliliter (pg/mL) range. IL-4 and IL-12p70 levels were undetectable in the majority of samples and were therefore excluded from further analysis. Mice exhibiting an osteopetrotic phenotype were also excluded, as excessive trabecular bone compromises bone marrow space, potentially impairing white blood cell production and immune function.

### Micro-computed tomography and skeletal analysis

Left hindlimbs of peri-vehicle (N = 15), peri-OF (N = 15), post-vehicle (N = 15) and post-OF (N = 5) mice were imaged by micro-computed tomography (μCT) using an Skyscan 1176 (Bruker, Belgium) performed at an isotropic voxel size of 4.98 μm. The X-ray tube was operated at 50 kV and 200 mA, and projections were collected using an exposure time of 960 ms every 0.6 ° over 180 ° rotation in combination with an 0.5 mm aluminium filter. Tomograms were reconstructed in NRecon (version 1.7.4.6; Bruker, Belgium). μCT images of calcium hydroxyapatite phantoms with mineral densities of 0.25 g/cm^3^ and 0.75 g/cm^3^ (Bruker, Belgium) were acquired using the same scan parameters and reconstructed as above for attenuation coefficient calibration prior to bone mineral density assessment(32, 33).

Ahead of morphometric analyses, reconstructed images underwent re-positioning in Dataviewer (version 1.5.6.2; Bruker, Belgium) to ensure consistent orientation and alignment for subsequent analyses. Segmentation of tibiae in repositioned images was performed in CTAn (version 1.23.0.2 +; Bruker, Belgium) and tibial length measured. 5% of the total bone length was used for 3D analyses of metaphyseal trabecular bone, beginning from the point at which the trabecular bridge visibly connects the primary spongiosa(34, 35) were segmented and binarized using a minimum threshold of 85. Morphometric parameters including bone volume/tissue volume (BV/TV), bone surface/tissue volume (BS/BV), trabecular number (Tb.N), trabecular spacing (Tb.Sp), trabecular thickness (Tb.Th), fractal dimension and bone mineral density (BMD) were measured in CTAn as described by Javaheri *et al.* (35). For cortical bone, a 2D slice-by-slice analysis approach was performed following segmentation using a minimum threshold of 80. Cortical morphometric parameters such as bone area (Ct.Ar), tissue area (Tt.Ar), bone area/tissue area (Ct.Ar/Tt.Ar), cortical thickness (Ct.Th), polar moment of inertia (J) and eccentricity, were then evaluated along the tibial length on a slice-by-slice basis. To exclude trabecular bone-rich regions in cortical analyses, 10% of the proximal and distal tibial length were excluded. Volume rendering of cortical and trabecular bone was performed in Avizo3D (version 2023.2; Thermofisher Scientific, USA).

### Western Blot

Protein from brain regions and liver was extracted in M-PER (Thermo Scientific, 78501) supplemented with Halt Protease and Phosphatase Inhibitor (Thermo Scientific, 78442) using a bath sonicator (Diagenode) run at 30 second increments. Protein concentration was determined using either Bradford Reagent (Bio-Rad, 5000205) or Pierce BCA Protein Assay (Thermo Scientific, 23225) and read at 560nm using the EnVision^®^ multimode plate reader (Perkin Elmer). 20 to 30 μg of protein was loaded on Criterion XT Precast Gels (Bio-Rad, 3450124) and run at 100–150V. Gels were transferred onto PVDF membranes (Bio-Rad, 1704157) using the Trans-Blot Turbo Transfer System (Bio-Rad, 1704150). Prior to probing with primary antibody, membranes were blocked at room temperature for 1 hour with either 5% milk (Bio-Rad, 1706404) or 5% BSA (Sigma, A9647) for phosphorylated proteins. Primary antibodies were incubated overnight at 4°C. Secondaries were incubated for 1 hour at room temperature. The following antibodies and concentrations were used: Phospho-Akt (Ser473) (D9E) XP Rabbit mAb (Cell Signaling, 4060) at 1:2,000; Akt (Cell Signaling, 9272) at 1:1,000; Phospho-S6 (Ser240/244) (Cell Signaling, 2215) at 1:1000; S6 (5G10) (Cell Signaling, 2217) at 1:10,000; Goat anti-Rabbit (Abcam, ab7090) 1:5,000. All antibodies were prepared in 5% BSA (Sigma, A9647) in TBS-T. Membranes were stripped using Restore Western Blot Stripping Buffer (Thermo Scientific, 21063) for 30 minutes at room temperature.

### General Statistics

Data are expressed as the mean ± SEM. Statistical analyses and graphing were performed with GraphPad Prism 8.2 (GraphPad Software; San Diego, CA, USA). An unpaired *t* test with Welch’s correction was used when comparing the means of two groups. When F-test of variance was significant (P < 0.05), the nonparametric Mann-Whitney test was used to compare two groups. One-way ANOVA with Tukey or Dunnett’s post-hoc analysis was used for multiple comparisons when more than two means were being compared. Two-way ANOVA with Sidak’s multiple comparison was used to test GTT and ITT curves over time. Inter-test correlations were analyzed with one-tailed Pearson correlation coefficient, and Bonferroni corrected based on the number of correlations compared. Adjusted p < 0.05 was deemed to be of statistical significance. Outliers were determined with ROUT outlier analysis, Q = 1%. For cortical bone analyses, Two-way ANOVA was used to compare between peri-vehicle, peri-VCD, post-vehicle and post-VCD groups using a bespoke code developed in R Studio (version 2024.12.0, build 467; R Foundation for Statistical Computing, Austria). Data normality was determined using the Shapiro-Wilk test. Data are presented as mean ± SEM and were considered statistically significant when p < 0.05 (34).

## Results

### VCD-treated mice had decreased ovarian follicles and changes in plasma hormone levels

**Figure 2:**
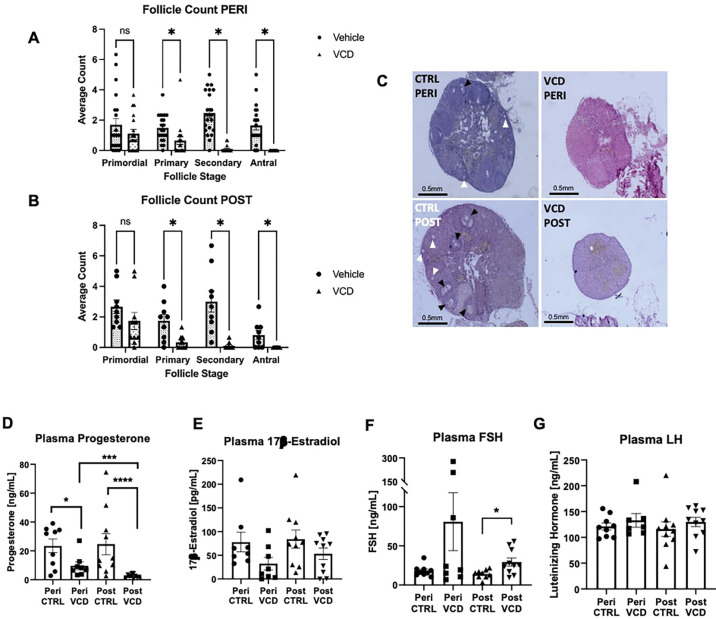
VCD-treated mice had decreased ovarian follicles at both peri- and post-OF time points. Average follicle counts per section of ovary at peri-OF (A) and post-OF (B) timepoints. C) Images of H&E-stained 5 μm ovary slices under each condition and timepoint at 4x magnification; black arrows point to antral follicles, white arrows point to secondary follicles. VCD-treated mice had decreased levels of circulating progesterone and increased levels of FSH. Plasma levels of progesterone (D), estradiol (E), FSH (F), and LH (G) measured with ELISA. Graph’s represent Mean ± SEM. Welch’s t-test or Multiple Mann-Whitney tests with multiple comparisons correction were used to compare means. *p<0.05, ***p<0.01, *** p<0.001, ****p<0.0001.

To characterize the impact of VCD treatment on ovarian status we analyzed ovarian follicles using H&E staining. VCD-treated mice had smaller ovaries at both time points compared to control mice (Fig. 2C). At both peri- and post-OF, VCD reduced primary, secondary, and antral follicles (Fig. 2A, 2B). Plasma levels of ovarian hormones progesterone and estradiol, and pituitary hormones FSH and LH were measured at the study endpoints. As expected, plasma progesterone was significantly lower in VCD-treated mice at both peri- (*t*(12.56) = 2.537, p = 0.0253) and post-OF (*U* = 3, p < 0.0001); and post-OF VCD-treated mice had lower levels that peri-OF VCD mice (*U* = 5, p = 0.0008) (Fig. 2D). Some VCD-treated mice had limited circulating estradiol at peri-OF (Fig. 2E). Note that available ELISA kit has 17.8% cross reactivity with estrone (E1), which is mainly produced in adipose tissue (36) and so complete loss of signal, even in post-OF was not expected (Fig. 2E). FSH, which is reported to increase post menopause, was greater in post-OF VCD mice (*t*(11.33) = 2.819, p = 0.0163) (Fig. 2F). No difference was seen between treatment groups or time points in plasma LH levels (Fig. 2G). Using Pearson’s correlation coefficient, plasma progesterone was negatively correlated with glucose tolerance test (GTT) AUC (*r* = −0.46, *p* = 0.0021) and GTT peak blood glucose (*r* = −0.29, *p* = 0.0434).

#### VCD-treated mice showed impaired glucose and insulin sensitivity.

**Figure 3:**
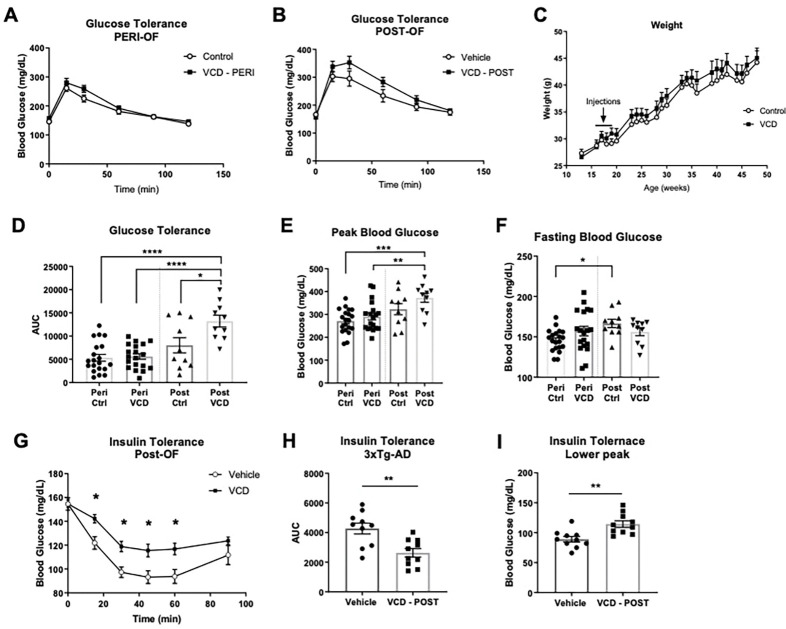
VCD-treated mice show decreased glucose sensitivity and insulin tolerance at post-OF. Intraperitoneal GTT results at peri-OF (A) and post-OF (B) female 3xTg-AD mice. C) Mouse weight overtime did not differ significantly between groups. Glucose tolerance area under the curve (D) and peak blood glucose (E) were significantly worse in post-OF VCD-treated animals. (F) Fasting blood glucose was significantly higher in control post-OF mice compared to at peri-OF timepoint. Insulin tolerance (G-I) was significantly reduced in post-OF VCD mice. Graph’s represent Mean ± SEM. One-way ANOVA with Tukey’s multiple comparison was used to compare means of groups of four. Welch’s t-test or Mann-Whitney test were used to compare means of two groups. *p<0.05, **p<0.01, ***p<0.001.

We measured i.p. glucose tolerance at peri- and post-OF timepoints (Fig. 3A, B). Glucose tolerance was significantly impaired in POST-OF mice compared to PERI-OF (*p* < 0.0001) and control mice (*p* = 0.0116) at the same timepoint (Fig. 3D). Peak blood glucose was greater in POST- compared to PERI-OF mice (*p* = 0.0083) (Fig. 3E). VCD-treated mice did not have altered body weight compared to control mice (Fig. 3C). Changes in glucose tolerance partly reflected impaired insulin sensitivity in POST-OF compared to control mice, with a reduced (glucose) area under the curve (*t*(17.03) = 3.516, *p* = 0.0026) and reduced peak (*t*(17.49) = 3.537, *p* = 0.0024) (Fig. 3G) in control mice. Changes in insulin sensitivity were not reflected in mammalian target of rapamycin (mTOR) status in the liver, as represented by the ratio of phosphorylated-Akt to Akt (mTORC2 signaling) and phosphorylated-S6 to S6 (mTORC1 signaling) (supplementary Fig. 1).

#### Proinflammatory Cytokine Levels are Elevated in Menopausal AD Mice.

**Figure 4:**
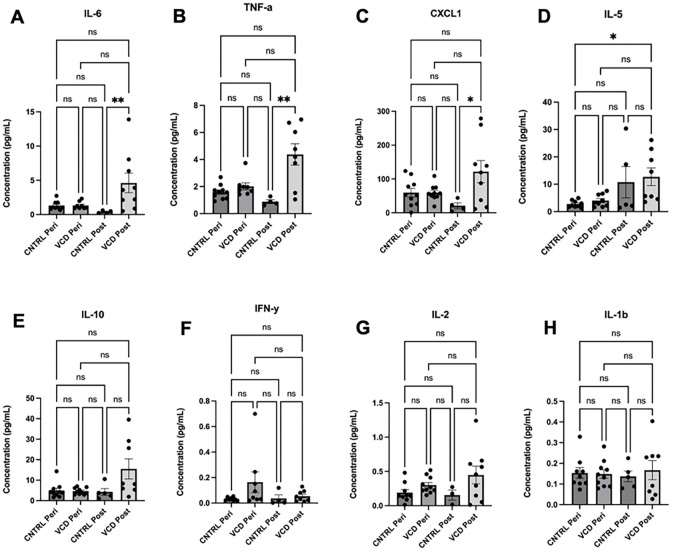
Aged VCD-treated mice display increased inflammatory signatures in plasma. Multiplex MSD chemiluminescence-based assay for eight proinflammatory cytokines at peri-Of and POST-OF time points (A-H). Plasma levels of IL-6 (A), TNF-α (B), and CXCL1 (D) were significantly increased in POST-OF mice compared to PERI-OF control mice of the same age. Plasma levels of IL-5 (C) were significantly increased in POST-OF mice compared to PERI- vehicle treated mice. Circulating cytokine levels for IL-10 (E), IFN-*γ* (F), IL-2 (G), and IL-1β (H) were not significant across groups. Graphs represent Mean ± SEM. Kruskal-Wallis test corrected for multiple comparisons was used to compare the means across the four groups. *p<0.05, **p<0.01.

To assess the impact of menopause on peripheral inflammation in an AD model, ten proinflammatory cytokines—IFN-γ, IL-1β, IL-2, IL-4, IL-5, IL-6, IL-10, IL-12p70, KC/GRO, and TNF-α—were measured in plasma using a multiplex MSD proinflammatory cytokine array. IL-4 and IL-12p70 levels were undetectable in the majority of samples and were therefore excluded from further analysis. Compared to vehicle-treated controls, menopausal AD mice showed no significant differences in plasma levels of IL-10 (*p* = 0.0632), IFN-γ (*p* = 0.1482), IL-2 (*p* = 0.1276), or IL-1β (*p* = 0.9791) (Fig. 4E–H). In contrast, post-menopausal mice exhibited significantly elevated levels of IL-6 (*p* = 0.018), TNF-α (*p* = 0.0023), and CXCL1 (*p* = 0.0256) compared to age-matched controls (Fig. 4A–C). Additionally, IL-5 levels were significantly higher in post-menopausal 3xTg-AD mice than in peri-menopausal controls (*p* = 0.0174) (Fig. 4D). This consistent increase in proinflammatory cytokines suggests that menopause contributes to enhanced peripheral inflammation in the AD mouse model.

### OF accelerates osteoporotic phenotype in 3xTg-AD mice

To explore links between menopause and AD, we evaluated the effects of VCD-induced ovarian failure on the skeletal phenotype in both trabecular and cortical bone compartments of the tibia. Initial screening of reconstructed microCT datasets in 3D showed that some tibiae exhibited a striking phenotype resembling osteopetrosis, featuring excessive trabecular bone throughout the metaphyseal and diaphyseal marrow space regions (Supplementary Fig. 2A, B). Intriguingly, the frequency of this osteopetrotic-like phenotype at the early peri-menopausal timepoint was 13% in tibiae from vehicle-treated mice (peri-vehicle) but was absent in mice subjected to VCD treatment (peri-OF). There was, likewise, a higher 50% frequency of this osteopetrotic phenotype in vehicle treated mice than in VCD treated mice (25%) at the more advanced experimental timepoint. Quantification of metaphyseal trabecular morphometric parameters in osteopetrotic-like tibiae confirmed the presence of significantly greater bone volume/tissue volume (BV/TV, *p* < 0.0001), bone surface/tissue volume (BS/TV, *p* < 0.0001), and bone mineral density (BMD, *p* < 0.0001) compared to non-osteopetrotic tibiae from the same treatment group and time point (Supplementary Table 1). In addition, trabecular number (Tb.N) was significantly higher while trabecular spacing (Tb.Sp, p < 0.0001) was significantly lower in osteopetrotic-like tibiae in the peri-vehicle, post-vehicle and post-VCD groups, while trabecular thickness (Tb.Th) was only significantly greater in osteopetrotic peri-vehicle tibiae versus healthy controls (p < 0.0001). In line with these findings, trabecular connectivity density (Conn.D) and fractal dimension were also significantly in osteopetrotic-like tibiae (*p* < 0.001), collectively implicating a mechanism in which osteoclast function and/or bone marrow homeostasis is influenced by the gradual depletion of female sex hormones. To focus on the staged effects of ovarian failure on bone mass and architecture, these osteopetrotic-like tibiae were excluded from subsequent analyses.

**Figure 5:**
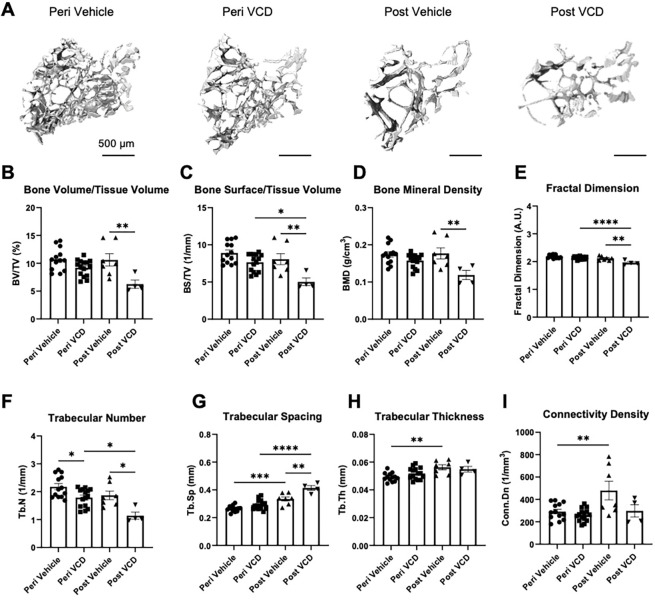
Tibial trabecular microarchitecture is modified by age and detrimentally affected by VCD-treatment at post-OF timepoint. Representative 3D reconstructions of the proximal tibia trabecular region (0–5% of tibia length) in control and VCD-treated mice at peri-OF and post-OF time points (A). μCT analysis of tibial bone volume/tissue volume (BV/TV, B), bone surface/tissue volume (BS/TV, C), bone mineral density (BMD, F), trabecular number (Tb.N, E), fractal dimension (F), trabecular spacing (Tb.Sp, G), trabecular thickness (Tb.Th, H) and tibia length (I). Data are presented as mean ± SEM with symbols representing individual animals. Statistical significance between groups was assessed using One-way ANOVA (P<0.05*, P<0.01**, P<0.001***, P<0.0001****).

3D inspection of all the non-osteopetrotic-like tibiae revealed prominent loss of metaphyseal trabeculae with age in vehicle-treated mice which was more pronounced in response to VCD exposure (Fig. 5A). Morphometric analyses revealed that long-term treatment with VCD (post-VCD versus post-vehicle) culminated in significant reductions in BV/TV (*p* = 0.007; Fig. 5B), BS/TV (*p* = 0.008; Fig. 5C), BMD (*p* = 0.006; Fig. 5D), fractal dimension (*p* = 0.001; Fig. 5E) and Tb.N (*p* = 0.014; Fig. 5F) and corresponding increases in Tb.Sp (*p* = 0.002; Fig. 5G) without significant modification of Tb.Th (Fig. 5H) or Conn.D (Fig. 5I). In vehicle-treated mice, both Tb.Sp (*p* = 0.0002) and Tb.Th (*p* = 0.003) showed significant increases over the course of the experiment (Fig. 5G and H, respectively). In contrast, short term treatment with VCD (peri-VCD) failed to induce changes to BV/TV (Fig. 5B), BS/TV (Fig. 5C), BMD (Fig. 5D), fractal dimension (Fig. 5E), Tb.Sp (Fig. 5G), Tb.Th (Fig. 5I) or Conn.D, while small but significant decreases in Tb.N (*p* = 0.04; Fig. 5F) were evident. More marked skeletal deficit was apparent in the VCD treated animals, whereby reductions in BS/TV (*p* = 0.011; Fig. 5C), fractal dimension (*p* < 0.0001; Fig. 5E), Tb.N (*p* = 0.016; Fig. 5E), in addition to greater Tb.Sp (*p* < 0.0001; Fig. 5G) were apparent over time. These data indicate that VCD-induced ovarian failure markedly modifies trabecular mass and architecture akin to an osteoporotic phenotype.

**Figure 6:**
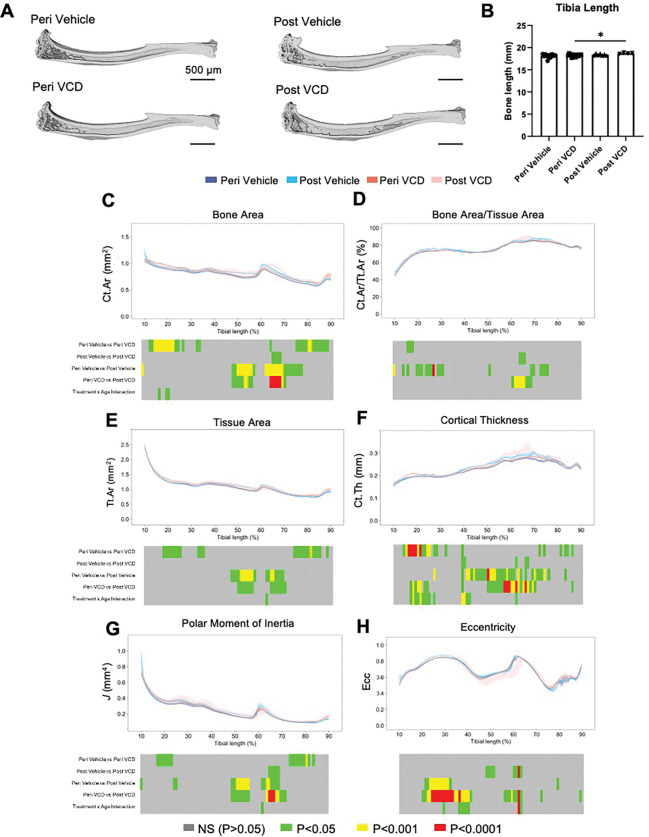
Ageing and VCD treatment produce spatial variations in cortical microarchitecture and tibial geometry. Representative 3D reconstructions of tibial cortex in mice treated with vehicle and VCD at the peri-OF and post-OF time points (A). Comparison of tibial length between groups (B). Bone area (Ct.Ar) (C), bone area/tissue area (Ct.Ar/Tt.Ar) (D), tissue area (Tt.Ar) (E), cortical thickness (Ct.Th) (F), polar moment of inertia (G) and eccentricity (H) were evaluated between 10% and 90% of the tibial length, excluding proximal and distal metaphyseal bone, and are presented as line graphs representing mean ± SEM. Graphical heat maps beneath show statistical differences at spatially matched locations along the tibial length with VCD treatment (peri vehicle vs. peri VCD and post vehicle vs. post VCD) and with age (peri vehicle vs. post vehicle and peri VCD vs. post VCD). Statistical significance between groups was assessed by one-way ANOVA (A; P<0.05*) and interaction between treatment (vehicle/VCD) and age (peri/post) assessed by two-way ANOVA (grey, P>0.05; green, P<0.05; yellow, P<0.001; red, P<0.0001).

Despite the pronounced changes to the trabecular bone in peri-OF and post-OF animals, gross anatomical deficits in the cortical bone architecture of tibiae were not visually discernible (Fig. 6A). Measurement of tibia length, however, revealed that prolonged treatment with VCD induces a subtle yet significant lengthening (Fig. 6B, *p* = 0.05). 2D cortical analyses, performed slice-by-slice revealed small regionally restricted changes in bone architecture in response to long-term VCD (post-vehicle versus post-VCD). These comprised increased bone area (Ct.Ar; Fig. 6C) and bone area/tissue area (Ct.Ar/Tt.Ar; Fig. 6D) restricted to regions distal to the tibiofibular junction (~ 65% of length); without compromising tissue area (Tt.Ar; Fig. 6E), complemented by a corresponding increase in cortical thickness (Ct.Th; Fig. 6F). Quantification of geometrical parameters, similarly, disclosed small, regionally restricted, increases in polar moment of inertia (Fig. 6G) and eccentricity (Fig. 6H) in response to long-term VCD treatment. In stark contrast, advancing age in vehicle treated animals (peri-vehicle vs. post-vehicle) led to more widespread elevations in both Ct.Ar and Tt.Ar (Fig. 6C and E), extending distally from the mid-diaphysis (~ 50–75%). Within this distal region, changes to Ct.Ar/Tt.Ar (Fig. 6D) were less pronounced, but were instead elevated proximally (~ 10–30%). Age-related increases in Ct.Th (Fig. 6F) were more extensive along the tibial length (~ 40–75%) while the polar moment of inertia was greater in regions corresponding to the mid-to-distal diaphysis (~ 50–70%) and eccentricity changes evident in the proximal tibia (~ 20–30%). In contrast to long-term VCD treatment, short-term VCD treatment resulted in increased Ct.Ar and Tt.Ar (Fig. 6C and 6E) in the proximal (~ 10–30%) and distal tibia (~ 75–90%), while corresponding increases in Ct.Ar/Tt.Ar were confined to the proximal tibiae (~ 15–20%; Fig. 6D). Increases in Ct.Th in response to short-term VCD treatment were also seen in the proximal (~ 15–40%) and distal tibia (~ 70–85%; Fig. 6F), where they contributed to elevations in the polar moment of inertia but not eccentricity (Fig. 6G and H, respectively). Age-related increases in Ct.Ar (Fig. 6C), Ct.Ar/Tt.Ar (Fig. 6D) and tissue area (Fig. 6E) were present proximal to the tibiofibular junction (~ 50–70%) in VCD-treated animals while widespread increases in Ct.Th (Fig. 6F) were present in regions spanning both the proximal tibia (~ 15–25%) and midshaft (~ 50–70%). These regionalized changes in cortical bone mass were linked to increases in the polar moment of inertia (Fig. 6G) and subtle alterations to eccentricity evident in the proximal tibia (~ 20–40%; Fig. 6H). Collectively, these data are consistent with spatially confined shifts in cortical bone mass and geometry in response to short and long-term VCD treatment that induce modifications to bone architecture.

## Discussion

Menopause marks the end of reproductive years for women and is characterized by the cessation of follicular maturation and decrease in gonadal hormones. These changes have impact in various diseases, including AD. While rodents do not go through menopause, menopause-like changes in endocrine state can be modeled either through ovariectomy or, as we show here, through ovotoxin-induced ovarian failure. Accelerated ovarian failure models have been used to explore menopause-linked disorders including metabolic syndrome (37), cardiovascular disease (27), and cognitive decline (38, 39). The aim of this study was to investigate how the VCD-induced OF model affects peripheral AD pathologies—specifically metabolic dysfunction, inflammation, and bone health—in the 3xTg-AD mouse. We report that menopause-like conditions exacerbate peripheral AD phenotypes, with the most dramatic differences observed in the post-menopausal time-point.

### Effects of ovarian failure on insulin sensitivity in 3xTg-AD mice

Metabolic disease is one of the most prevalent risk factors for AD (40), and menopause increases the risk of metabolic disease in women (41, 42). In the present study we show that OF has a negative effect on glucose and insulin tolerance at post-OF, similar to a study in C57B6/J mice treated with VCD which had impaired glucose tolerance at 6 months following VCD injection, but not at 4.5 months (43). Of note, the 3xTg-AD mouse has age-dependent impairments in glucose tolerance starting at 10 months (44). Our study supports the notion that loss of ovarian hormones exacerbates age-related glucose intolerance. Reduced glucose tolerance has also been linked to worsening of neurological outcomes in AD mice. Pre-diabetic AD mice, modeled in APP/PS1 mice fed a high fat diet, had significant neuronal loss, and APP/PS1 mice crossed with an obesity driven type 2 diabetes model (db/db) had even greater neuronal loss and decreased synaptic density (45), supporting the link between metabolic dysfunction and cognitive decline. Impaired insulin sensitivity is also associated with AD, with ~ 25% of people with Type 2 Diabetes (T2DM) being severely insulin resistant. Overall, T2DM is associated with a doubling of the risk for AD (40). The offspring of AD mice bred with insulin resistant mice exhibited cognitive impairment at an earlier age than AD mice lacking insulin resistance (46). This was accompanied by an increase in amyloid burden and appeared to be mediated by a downregulation in nicotinic acetylcholine receptor. Interestingly, the connection between T2DM and AD is not considered to reflect an increase in Aβ or neurofibrillary tangles, but rather increased systemic inflammation leading to more microvascular infarcts (47). Our findings support the paradigm that OF can increase the risk of metabolic disease and peripheral inflammation, all of which may exacerbate AD-linked processes.

### OF increases peripheral inflammation in AD mice

The loss of estrogen during menopause is also known to promote a pro-inflammatory state, both centrally and peripherally, by modulating immune response (48). Independently, inflammation is associated with both aging and AD risk, with several studies identifying heightened levels of pro-inflammatory cytokines in AD patients compared to neurologically normal controls (20, 49, 50). Consistent with these findings, our study demonstrates that post-menopausal AD mice exhibit elevated circulating levels of pro-inflammatory cytokines relative to AD mice without OF. These findings suggest that menopause associated OF amplifies peripheral inflammation in the context of AD, which may contribute to the exacerbation of AD pathology. Specifically, we observed increased levels of the pro-inflammatory cytokines IL-6, TNF-α, and CXCL1, all of which have been implicated in cognitive impairments associated with AD (51–53). Notably, our findings reinforce prior reports linking elevated IL-6 and TNF-α to disruptions in peripheral metabolic processes, specifically through interference with insulin signaling and activation of cellular stress pathways (53, 54). These effects highlight a potential mechanism by which systemic inflammation, exacerbated by menopause, may contribute to AD pathophysiology.

### Menopause Accelerates Osteoporotic Phenotype in 3xTg-AD Mice

Estrogen deficiency arising due to menopause is a well-established driver of microarchitectural deterioration and loss of bone mass that predisposes to fragility fractures(55). Herein, advancing age alone resulted in small but statistically insignificant reductions in trabecular BV/TV, BS/BV and Tb.N alongside significant trabecular thickening and greater spacing, which aligns with prior reports of age-related trabecular degeneration in mice(56). As expected, these age-associated changes were exacerbated by prolonged VCD exposure when marked reductions in BV/TV, BS/TV, BMD, fractal dimension and Tb.N and increased Tb.Sp akin to changes found in aging humans(57, 58) ovariectomized mice(59, 60) and VCD-treated mice(61). Interestingly, short-term exposure to VCD at the peri-OF stage resulted in modest compromise in Tb.N, whereas the transition from peri-OF to post-OF resulted in progressive structural trabecular decline.

Cortical bone also underwent significant structural adaptations in response to VCD treatment. In contrast to the deficits observed in the trabecular compartment, these changes were however restricted to specific regions of the tibial cortex. In the post-OF group, long-term exposure to VCD elicited localized increases in Ct.Ar, Ct.Ar/Ct.Tt, Ct.Th, polar moment of inertia and eccentricity in the distal tibia independent of regionalized tissue area expansion. In vehicle-treated tibiae, aging alone was associated with widespread expansion of Ct.Th and Tt.Ar along the tibial shaft without impacting tibial geometry. Conversely, short-term VCD induced cortical thickening in the distal and proximal tibia that coincided with increases in Ct.Ar, Tt.Ar and Ct.Ar/Tt.Ar and polar moment of inertia while eccentricity was preserved. This contrasted with changes in the cortices of VCD-treated mice where aging induced region-specific expansion of Ct.Ar, Tt.Ar, Ct.Ar/Tt.Ar and Ct.Th with large-scale changes to cortical geometry across the tibial shaft. Although OVX is classically associated with cortical thinning, marrow space expansion, and loss of mechanical integrity(59, 62), several studies have demonstrated that estrogen deficiency can transiently induce cortical expansion through periosteal apposition. In rats, OVX has been shown to induce increased Ct.Ar and Ct.Th at specific diaphyseal sites(63). Further studies have reported that such cortical adaptations are age and site-dependent, with periosteal formation reported to compensate for early resorptive bone loss leading to enhanced polar moment of inertia while conserving bending strength(64, 65). These observations align with our findings where the regionalized increases in Ct.Ar, Ct.Th and polar moment of inertia likely reflect an adaptive periosteal response to gradual ovarian failure. Importantly, it has been reported that the magnitude of cortical bone loss post-OVX is influenced by baseline morphology, with several mouse strains that possess high baseline bone mass exhibiting a protection against the detrimental effects of estrogen loss on cortical bone(59). While the age-related increases in Ct.Ar we observed in vehicle-treated animals are consistent with the 2005 findings of Li *et al*. (62), the regionalized enhancement of cortical parameters after VCD exposure suggest that gradual ovarian failure does not prevent age-related cortical bone changes from occurring.

This partial preservation and spatial restriction of age-related effects on cortical bone may help explain the discrepancies between our observations and earlier findings(60, 66). An alternative hypothesis is that this gradual decline in ovarian function may provide sufficient time for cortical, but not trabecular, bone adaptation through periosteal apposition to offset endosteal bone loss, leading to a net gain of cortical bone in specific tibial regions. Collectively, indicating that VCD-induced ovarian failure in the 3xTg-AD model does not recapitulate the cortical deterioration commonly reported in OVX models. Instead, the pattern of regionalized adaptations to cortical bone mass and geometry suggests a more complex and potentially protective bone response to transitioning sex hormone levels which requires further attention in future studies.

The bone phenotypes nonetheless coincide with clear endocrine changes provoked by VCD-induced ovarian failure in 3xTg mice. Our histochemical analyses indeed confirmed the pronounced loss of primary, secondary, and antral follicles at both peri-OF and post-OF stages was accompanied by a significant reduction in plasma progesterone, elevated FSH, and modest fluctuations in 17β-estradiol, consistent with previous studies characterizing the effects of VCD-induced OF(60, 67–69). The mechanism underlying menopause related bone loss is thought to involve the gradual decline in 17β-estradiol production through the promotion of osteoclastogenesis, both directly and indirectly through upregulation of pro-inflammatory cytokines such as IL-6 and TNFα(70, 71). Similarly, FSH has been shown to enhance osteoclast differentiation and negatively regulate bone (72), whereas progesterone, like estrogen, exerts osteoanabolic effects(73). Notably, residual follicle-depleted tissue from VCD-treated mice has been shown to be capable of producing androstenedione(67), a steroid shown to protect against accelerated bone loss following ovariectomy(60, 66). Circulating androstenedione has been shown to be higher in young VCD-treated mice compared to those cycling controls gradually declining with the age-related loss of ovarian function(74). Although androstenedione levels were not measured in our study, its known protective effects on bone may in part explain the conceivably mild trabecular deficits and protection of cortical changes observed at the peri-OF stage. It’s presumed age-related decline coupled with reduced progesterone and elevated FSH are likely to act in concert to promote the severe trabecular and cortical bone changes observed at the post-OF stage. Importantly, our data also revealed that these menopause-associated skeletal shifts were associated with peripheral inflammation inherent to AD, which may function synergistically to promote osteoclastogenesis to further accelerate bone loss. Thus, the combined consequences of follicular depletion and hormone loss, along with heightened systemic inflammation, offers mechanistic explanation for the increased risk of osteoporosis in postmenopausal women, particularly those that have or are at risk of AD.

## Conclusion

In summary, we find that OF in the 3xTg-AD mouse enhances established AD pathological processes including metabolic dysfunction, peripheral inflammation, and osteoporotic bone phenotype, but in a more subtle manner than ovariectomized studies. In contrast to previous studies using accelerated OF, which begin VCD treatment around post-natal day 50, our study began treating mice around post-natal day 120 as we wanted to observe the interaction between age and OF. Notably, AD pathology in 3xTg-AD mice does not emerge until 6 months of age or later. The independent importance of the interactions between age and OF will need to be investigated in larger studies. Nevertheless, those events we did observe had a clear and compelling role in molecular mechanisms linked to AD. Collectively, our findings support the hypothesis that altered gonadal hormone production during menopause contributes to disrupted metabolic homeostasis, heightened peripheral inflammation, and an accelerated osteoporotic phenotype; factors that may converge to increase Alzheimer’s disease risk.

Intriguingly, a subset of 3xTg-AD mice displayed a pronounced osteopetrotic-like tibial phenotype, characterized by dense and disorganized trabecular bone throughout the marrow space(75–77). This aberrant phenotype, likely driven by impaired bone resorption, was seen only in vehicle-treated mice at the younger age (absent in VCD-treated), increased to 50% in older vehicle-treated mice, and was less frequent (25%) in VCD-treated mice at the post-OF timepoint. Trabecular morphometric analyses confirmed the presence of significantly elevated BV/TV, BS/TV, BMD, Tb.N and Conn.D along with lower Tb.Sp across all animals harboring this osteopetrotic phenotype; raised fractal dimension and Conn.D similarly indicate a more complex trabecular network consistent with a disturbance to osteoclast-mediated resorption. The emergence of this phenotype in the presence of functioning ovaries, and the partial rescue by VCD treatment suggests that the pro-inflammatory milieu characteristic of the 3xTg-AD model(78) may disrupt osteoclast function in a subset of animals leading to the pathological retention of trabeculae within the tibia. This aligns with previous findings linking AD-related neuroinflammation and systemic immune dysregulation to altered peripheral bone turnover and cognitive decline(79). VCD-induced attenuation of this phenotype may highlight a regulatory role for high FSH/low progesterone in the restoration of osteoclast function and thus the re-establishment of skeletal homeostasis. Together, these data highlight the complex interplay between neuroinflammatory status, ovarian function, and bone architecture in the context of AD.

## Supplementary Material

Supplementary Files

This is a list of supplementary files associated with this preprint. Click to download.

• Manuscriptsuppfigure2.jpeg

• Manuscriptsuppfigure1.jpeg

• Manuscriptsupptable1.jpeg

## Data Availability

All data generated or analyzed during this study are included in this published article (and its supplementary information files).

## Abbreviations

AD: Alzheimer’s disease
Aβ: Amyloid-beta
VCD: 4-vinylcyclohexene diepoxide
OV: Ovarian failure
FSH: Follicle-stimulating hormone
LH: Luteinizing hormone
3xTg-AD: Triple transgenic Alzheimer’s disease
D.P.I.: Days post injection
GTT: Glucose tolerance testing
ITT: Insulin tolerance testing
PFC: Prefrontal cortex
ERC: Entorhinal cortex
H&E: Hematoxylin and eosin
μCT: Micro-computed tomography
BV: Bone volume
TV: Tissue volume
BS: Bone surface
Tb.N: Trabecular number
Tb.Sp: Trabecular spacing
Tb.Th: Trabecular thickness
BMD: Bone mineral density
Ct.Ar: Cortical area
Tt.Ar: Tissue area
Ct.Th: Cortical thickness
J: Polar moment of inertia
E1: Estrone
mTOR: Mammalian target of rapamycin
Conn.D: Connective density
T2DM: Type 2 Diabetes
